# NEMA NU 2–2018 performance evaluation of a new generation 30-cm axial field-of-view Discovery MI PET/CT

**DOI:** 10.1007/s00259-022-05751-7

**Published:** 2022-03-14

**Authors:** Konstantinos G. Zeimpekis, Fotis A. Kotasidis, Martin Huellner, Alexandra Nemirovsky, Philipp A. Kaufmann, Valerie Treyer

**Affiliations:** 1 Department of Nuclear Medicine, University Hospital Zurich, University of Zurich, Rämistrasse 100, 8091 Zurich, Switzerland; 2 Department of Nuclear Medicine, Inselspital, Bern University Hospital, University of Bern, Freiburgstrasse 18, 3010 Bern, Switzerland; 3 GE Healthcare, Waukesha, WI USA

**Keywords:** PET/CT, NEMA NU 2, 6-Ring, Discovery MI 6-ring scanner, Performance evaluation

## Abstract

**Purpose:**

The DMI PET/CT is a modular silicon photomultiplier–based scanner with an axial field-of-view (FOV) between 15 and 25 cm depending on ring configuration (3, 4, or 5 rings). A new generation of the system includes a reengineered detector module, featuring improved electronics and an additional 6th ring, extending the axial FOV to 30 cm. We report on the performance evaluation of the 6-ring upgraded Generation 2 (Gen2) system while values are also reported for the 5-ring configuration of the very same system prior to the upgrade.

**Methods:**

PET performance was evaluated using the NEMA NU 2–2018 standard for spatial resolution, sensitivity, image quality, count rate performance, timing resolution, and image co-registration accuracy. Patient images were used to assess image quality.

**Results:**

The average system sensitivity was measured at 32.76 cps/kBq (~ 47% increase to 5 rings at 22.29 cps/kBq) while noise equivalent count rate peaked at 434.3 kcps corresponding to 23.6 kBq/mL (~ 60% increase to Generation 1 (Gen1) and 39% to Gen2 5 rings). Contrast recovery ranged between 54.5 and 85.8% similar to 5 rings, while the 6 rings provided lower background variability (2.3–8.5% for 5 rings vs 1.9–6.8% for 6 rings) and lower lung error (4.0% for the 5 rings and 3.16% for the 6 rings). Transverse/axial full width at half-maximum (FWHM) at 1 cm (3.79/4.26 mm) and 10 cm (4.29/4.55 mm), scatter fraction (40.2%), energy resolution (9.63%), and time-of-flight (TOF) resolution (389.6 ps at 0 kBq/mL) were in line to previously reported values measured across different system configurations. Improved patient image quality is obtained with the 6 rings compared to the 5 rings, while image quality is retained even at reduced scan times, enabling WB dynamic acquisitions.

**Conclusions:**

The higher sensitivity of the 6-ring DMI compared to the 5-ring configuration may lead to improved image quality of clinical images at reduced scan time. Additionally, it could equally be used to allow improved temporal sampling and/or reduced overall scan time in dynamic acquisitions. Conversely, temporal sampling and scan time could be traded per application to further drive injected dose at lower levels.

## Introduction

Hybrid positron emission tomography/computed tomography (PET/CT) imaging has been the spearhead of the nuclear medicine since its introduction in the early 2000s. During recent years, there have been important technological improvements, both in hardware and software that enhanced detection efficiency and image quality, as well as improved patient comfort [1, 2]. The latest one is the utilization of digital silicon photomultiplier (SiPM) detectors [2] giving rise to a new era of PET/CT scanners.

Recently, a new generation of the DMI modular system has been introduced (DMI Gen2) in which the detector module (dmod) has been reengineered to house an additional detector unit, allowing a 6th ring detector configuration to be added in the existing ones and extending the maximum axial FOV to 30 cm. Additional modifications to the dmod-integrated circuits for faster coincidence event processing allow for improved count rate performance on all pre-existing and new extended axial FOV detector configurations. Incorporation of an additional ring coupled with improved dmod photon-counting electronics is expected to substantially improve the intrinsic sensitivity as well as count rate capability of such extended axial FOV system. Such a change in detector specifications and corresponding performance is expected to impact upon image quality, scan time, and injected dose in static and dynamic acquisitions performed. Therefore, evaluating the performance of the system is of importance to guide future workflow and protocol optimization as well as image quality acquisition and reconstruction parameters. A 5-ring 25-cm Gen2 system was installed in the department of nuclear medicine at the University Hospital Zurich, which subsequently was upgraded to include the additional 6th ring, thereby extending the axial FOV to 30 cm.

In this work, we report on the National Electric Manufacturer’s Association (NEMA) [3] performance evaluation measurements, performed on a 6-ring Gen2 DMI system while measurements are reported for and compared with the 5-ring configuration of the very same system. The performance assessment followed the latest iteration of the NEMA guidelines (NU 2–2018) [3] which succeeded the NU 2–2012 [4] and which include tests on spatial resolution, sensitivity, image quality, count rate performance, count losses and correction accuracy, PET/CT image co-registration, energy, and time resolution.

## Materials and methods

### Evaluation of PET performance

All tests were performed at the University Hospital of Zurich following the NEMA NU 2–2018 standards [3] and the results were generated using software tools provided by the manufacturer. An energy window of 425–650 keV was used along with all necessary corrections (e.g., attenuation, decay, scatter, and randoms). Detector calibrations, well counter corrections, table characterization, and daily quality assurance (QA) were all performed immediately prior to commencing any NEMA testing. All activities of ^18^F used during the tests were measured on an IBC dose calibrator (Comecer S.p.A) which was calibrated according to the standards of the Swiss Federal Institute of Metrology METAS [5].

### Scanner characteristics

The GE Discovery MI Gen1 PET/CT [6] is a hybrid SiPM-based scanner with dmod of 3 axial field-of-view (FOV) configurations (3 rings [15 cm] [7], 4 rings [20 cm] [8], and 5 rings [25 cm] [9]). Each of the 34 dmods consists of 3, 4, or 5 detector units within each of which are housed 16 × 9 lutetium-based crystals (3.95 mm × 5.3 mm × 25 mm) arranged in four 4 × 9 blocks. Each ring is comprised of 136 blocks while each block is coupled to three 3 × 2 grid arrays of Hamamatsu SiPMs with a segmented light guide between the SiPM ASIC and scintillator. A water-cooling system keeps the SIPMs to a temperature of approximately 19 °C with real time local temperature readout.

### Spatial resolution

Three ^18^F point sources with activity concentration > 500 MBq/mL were prepared and used to measure the spatial resolution. ^18^F drops were suspended in a flat tray and subsequently drawn and sealed into capillary tubes, with the drops being less than 1 mm in the axial direction. The tubes were placed inside a phantom holder latched into the accessory slot at the front of the cradle and positioned at 1 cm, 10 cm, and 20 cm from the isocenter. Following an initial scan to verify accurate positioning to within ± 1 mm, the point sources were imaged first at the center and then at 1/8th of the axial FOV for 1 min, respectively. The acquired data were reconstructed using VuePoint HD (VPHD) non-time-of-flight (TOF)-ordered subset expectation maximization (OSEM) without resolution modeling with 4 iterations/34 subsets and 2-mm Gaussian filtering, filtered back-projection (FBP), and QClear with beta 20 (BSREM with resolution modeling) on a 384 × 384 matrix. Results are reported for full width at half-maximum (FWHM) and tenth-maximum (FWTM) in the radial, tangential, and axial direction.

### Sensitivity

The sensitivity test measures the counts per second that the scanner measures for every unit of activity. The test is ran at low activity levels, in which count losses are negligible. A 70-cm long 2.3-mL line was filled with 3.87 MBq of ^18^F at scan time and placed successively inside aluminum sleeves of ever-increasing attenuating material for a total of 5 acquisitions of 1 min each, with the results extrapolated to give the scanner sensitivity with no attenuating material. The line was prepared using higher activity levels, in order to achieve higher accuracy from the dose calibrator and left to decay in order to reduce deadtime and random contributions. Sensitivity measurements are given for true-only events after random subtraction. The line together with the sleeves were placed on a phantom holder and the phantom assembly was positioned precisely at the center of the transverse FOV and 10 cm off the isocenter, using sinogram qualitative and quantitative assessment, the gantry’s laser system, and a tubular spirit level.

### Count rate performance

To measure count rate performance of the scanner across a range of radioactivity levels, a long acquisition was performed starting from a high activity level. A 70-cm long 5.15-mL line filled with 834 MBq of ^18^F at scan start was threaded inside a 70-cm polyethylene phantom. The polyethylene phantom was suspended on shims, with the center of the phantom aligned to the scanner center and the line closest to the cradle’s surface. The acquisition was divided in 24 frames covering ever reducing activity concentration levels and with 500 k events per frame. We report on recorded prompts, randoms, trues, scatter, and noise equivalent counts (NEC) relative to the activity in the FOV. The peak trues and the noise equivalent count rates (NECRs) were calculated at their corresponding activity concentrations as well as scatter fraction at peak NECR. The scatter fraction across the same activity levels is also measured and reported.

### Count loss correction and accuracy

Data from the count rate test were used in this test by comparing the true rate calculated using count loss and random corrections with the true rate extrapolated from measurements with negligible count losses and randoms. We report on the relative count error as well as maximum absolute relative error below peak NECR.

### Image quality

Image quality was assessed with the NEMA international electrotechnical commission (IEC) body phantom. The phantom contains a lung insert in the middle, and six fillable spheres with increasing diameter (10, 13, 17, 22, 28, and 37 mm). The lung insert was filled with Styrofoam with an average density of 0.3-g/mL simulating lung tissue. The background was filled with 5.3 kBq/mL of ^18^F at scan time while all spheres were hot (as opposed to the 2 largest being cold in NEMA NU 2–2012) with an activity concentration of 4:1. To simulate activity outside the FOV, a scatter phantom was placed behind the body phantom with ~ 120 MBq at scan time. Three consecutive scans were performed to improve reproducibility. Scan times were chosen to emulate a 100-cm 30-min whole-body scan. The scan durations were 6:23 min, 6:39 min, and 6:54 min accordingly for the 6-ring configuration and 5:23, 5:34, and 5:46 min for the 5-ring configuration. Acquisitions were progressively longer to account for radioactivity decay between the 3 consecutive scan realizations and keep similar counting statistics.. Images were reconstructed using TOF OSEM 4 iterations/34 subsets 2-mm Gaussian (VPFX) and BSREM beta 50/no filtering (QClear) and a 384 × 384 image matrix. Regions-of-interest were automatically drawn on the background and spheres, and contrast recovery, background variability, and average lung error were measured according to NEMA specifications.

### Energy and timing resolutions

The data from the count rate performance test were also used to measure the system’s timing resolution. The time resolution was calculated as the FWHM of the time distribution of coincident events after correcting for scatter, random, and off-center source position. Measurements are reported at various activity levels below the peak NECR. Energy resolution was measured by a line source positioned at the center of FOV.

### PET/CT co-registration

PET/CT co-registration accuracy measures the co-registration error between the 2 subsystems and was assessed using 3 of the IEC phantom spheres (27 mm, 33 mm, and 28 mm) at 2 locations within the PET and CT field-of-view. A weight of 115 kg and simulating a patient, was distributed evenly (57.5 kg) over the cradle at 2 regions 65 cm each and spaced 30 cm apart. The phantom holder was axially positioned at 5 cm and at 100 cm from the edge of the cradle in accordance to NEMA guidelines. To prepare the phantom, a mixture of 60 MBq of ^18^F and CT contrast medium was used to fill the spheres, which subsequently were mounted on a phantom holder at fixed locations [(0,1), (20,0), and (0,20)] in the transaxial FOV. The phantom was imaged for 3 min in each of the 2 positions and the data were reconstructed with OSEM (4 iterations/34 subsets). The centroids of the spheres were calculated from the PET and CT data, and the co-registration error was determined by calculating the distance between the centroids.

### Patient imaging

For qualitative assessment of image quality, clinical imaging example datasets from patients were used for visualization. The study has been approved by the institutional review board and all subjects signed an informed consent form. A single-patient dataset acquired on the DMI Gen2 first with the 5-ring system configuration and later with the 6-ring configuration was retrospectively analyzed. The patient was injected with ~ 290 MBq of ^18^F-FDG, and data were acquired for 10 min with 5-bed positions on the 5-ring system (94 cm axial coverage) and 4-bed positions on the 6-ring system (96 cm axial coverage), allowing a representative qualitative assessment across the 2 configurations. Additionally, list mode data from an ^18^F-PSMA patient dataset (3 MBq/kg − 240 MBq in total) acquired on the 6-ring system were also retrospectively replayed, and reconstructed at ever decreasing scan durations (150 s/bed – 60 s/bed). Finally, a single-patient dataset acquired at different times post-injection with a dedicated WB dynamic protocol (Dynamic IQ, GE Healthcare) is also shown. The patient was injected with 2.6 MBq/kg (total of 222 MBq) and scanned with 50 s/bed for 8-bed positions. Reconstructed images are shown for 4 dynamic frames at 11 min, 25 min, 39 min, and 54 min post-injection.

## Results

### Performance evaluation

**Fig. 1:**
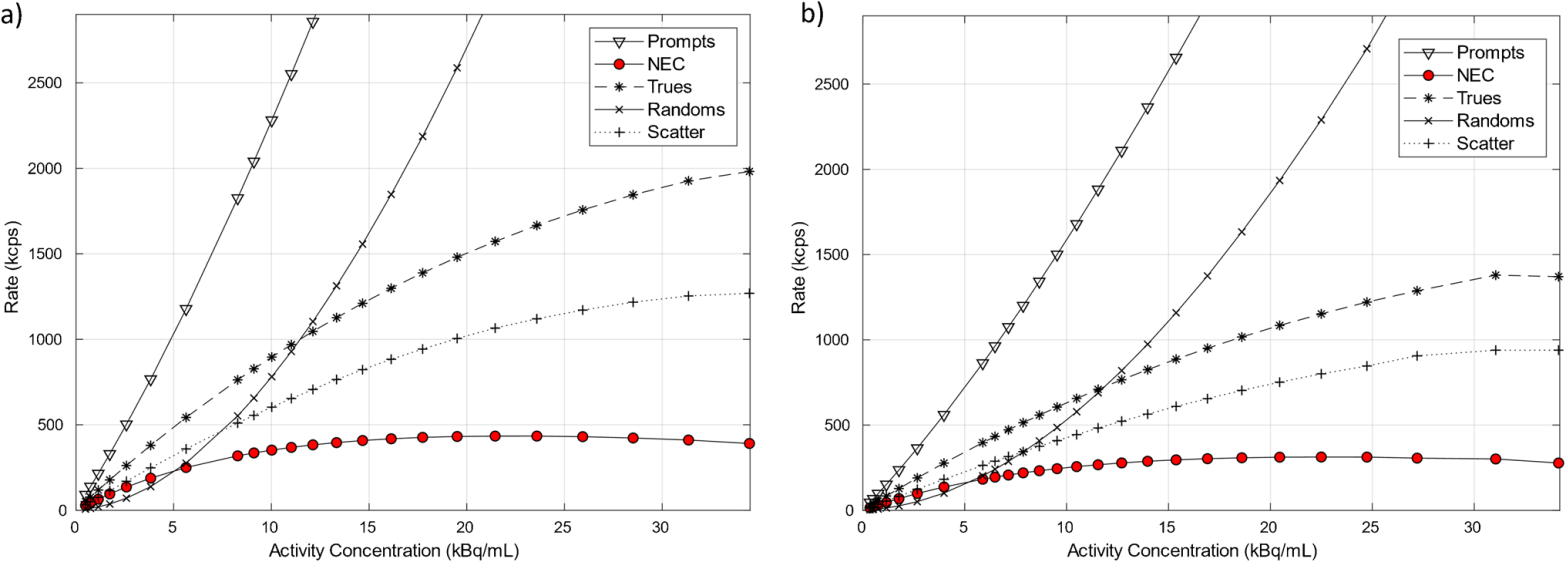
Count rate curves for prompts, randoms, trues, scatter, and noise equivalent counts for the 6-ring Gen2 DMI system (**a**) and for the 5-ring configuration of the same system (**b**). Peak NECR on the 6-ring system was measured at 434.3 kcps at 23.6 kBq/mL while the 5-ring NECR peaked at 312.9 kcps at 22.5 kBq/mL

**Fig. 2:**
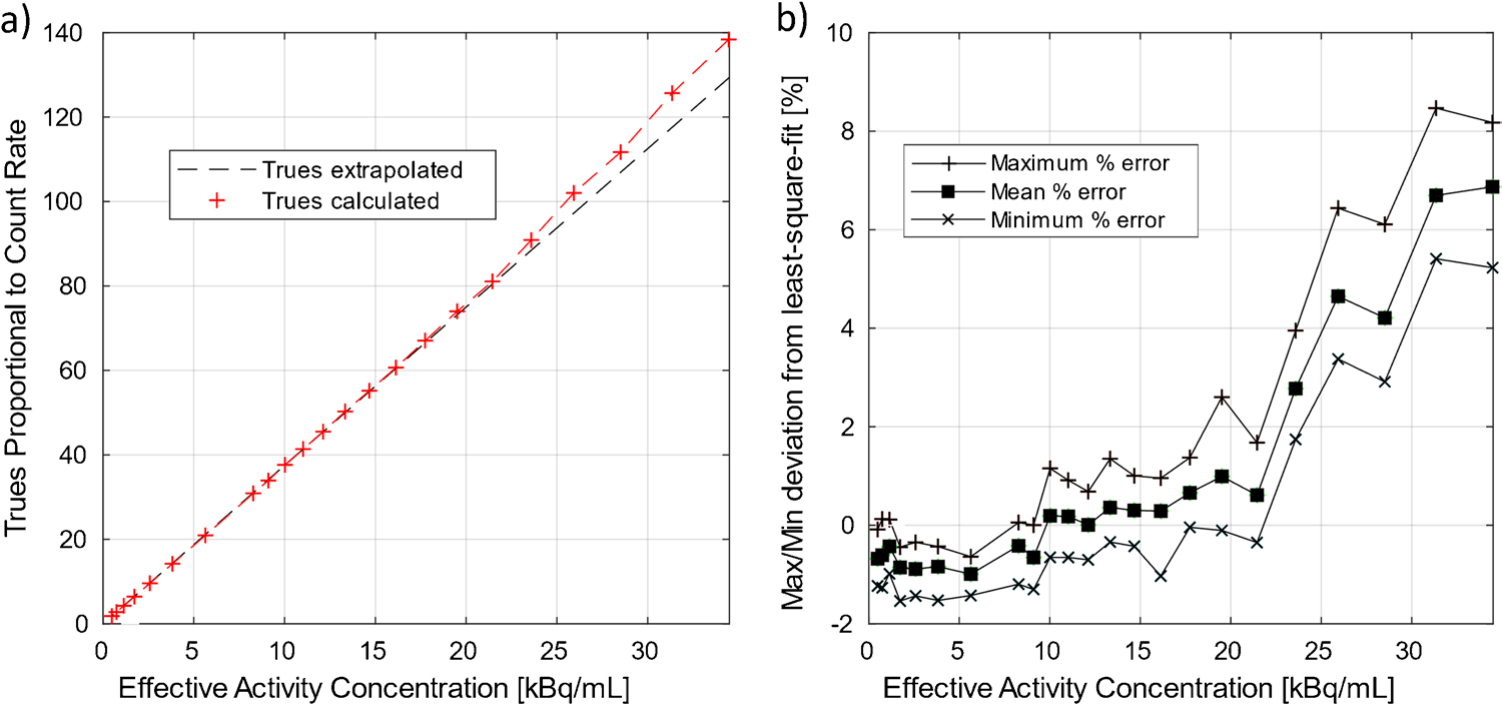
Corrected over extrapolated true rate (**a**) at various activity concentration levels for the 6-ring Gen2 DMI system together with the minimum, maximum, and mean deviation (**b**) between them. Maximum absolute error and mean error below peak NECR were measured at 3.95% and 2.77%, respectively. Due to similarity between system configurations, only the 6-ring system results are presented

**Fig. 3:**
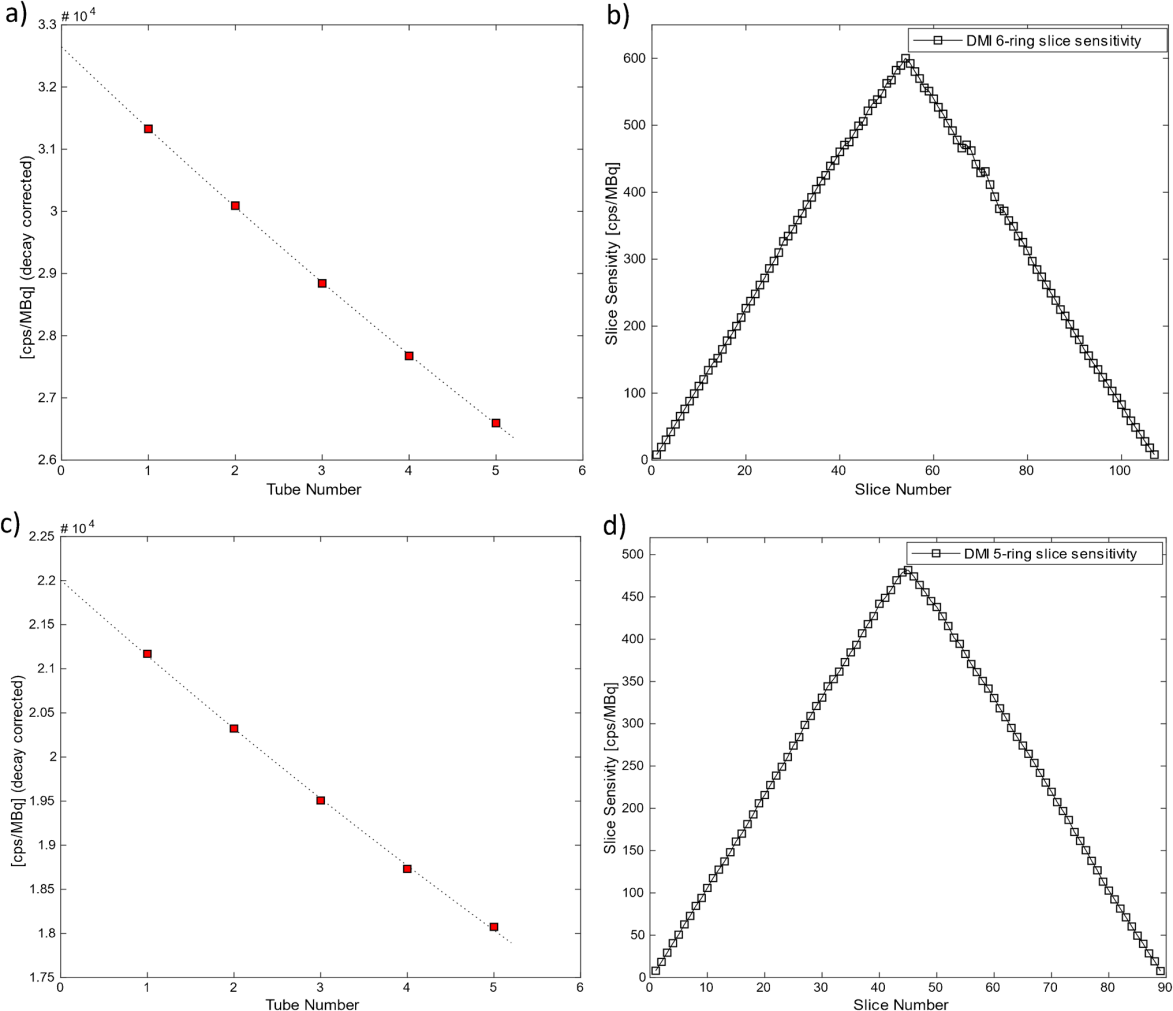
Sensitivity measurements at 0 cm offset across the 5 aluminum sleeves extrapolated to zero attenuating material (**a**, **c**) and the slice sensitivity profile across the axial FOV (**b**, **d**) for the 6-ring Gen2 DMI system (**a**, **b**) and 5-ring configuration of the same system (**c**, **d**). Sensitivity was measured at 32.88 cps/kBq and 22.01 cps/kBq, respectively

Radial, tangential, and axial FWHM measurements for both configurations can be found in Table 1. Spatial resolution results between the 5-ring and 6-ring configurations were similar. With respect to count rate performance, the peak NECR on the 6-ring system was measured at 434.3 kcps at 23.6 kBq/mL while in comparison the 5-ring NECR peaked at 312.9 kcps at 22.5 kBq/mL (Fig. 1). Using the same measurements, true count rate accuracy was assessed and is given in Fig. 2. Maximum absolute error and mean error below peak NECR was measured at 3.95% and 2.77% for the 6-ring and 4.61% and 2.31% for the 5-ring Gen2 respectively and with a linear response for a wide range of activity concentrations up to peak NECR. Sensitivity was measured at 32.64 cps/kBq and 32.88 cps/kBq at the center and at 10 cm radial offset (average 32.76 cps/kBq) while, in comparison and prior to the ring upgrade, sensitivity of the 5-ring configuration was found to be 22.01 cps/kBq and 22.59 cps/kBq, (average 22.3 cps/kBq) which is in line with previously reported values. Sensitivity measurements across the 5 aluminum sleeves extrapolated to attenuation-free value, as well as the slice sensitivity profile, are shown in Fig. 3. Image quality for the 6-ring configuration was assessed using the IEC phantom across the 6 spheres and the contrast recovery, background variability, and lung error ranged between 54.5–85.8%, 1.9–6.8%, and 3.16% respectively for OSEM, and 66.4–89.6%, 1.6–4.8%, and 2.6% respectively for QClear. For the 5-ring Gen2, the contrast recovery and background variability for OSEM ranged between 48–86.9% and 2.3–8.5% respectively with a lung error of 4%, while for QClear, it ranged between 64.5–90.2% and 1.8–6.5% respectively with a lung error of 3.2%.

**Table 1:** NEMA NU 2–2018 results across all performed tests for the 30-cm aFOV DMI Gen2 6-ring system together with the 5-ring configuration results acquired prior to the ring upgrade

Parameter		
System	DMI Gen2 5R	DMI Gen2 6R
Axial/transaxial FOV (cm)	25/70	30/70
Scintillator size (mm × mm × mm)	3.95 × 5.3 × 25	3.95 × 5.3 × 25
Spatial resolution (VPHD)		
1 cm radial/tangential/axial FWHM (mm)	3.84/3.89/4.33	3.72/3.87/4.26
10 cm radial/tangential/axial FWHM (mm)	4.88/3.88/3.93	4.80/3.79/4.55
20 cm radial/tangential/axial FWHM (mm)	7.15/4.45/4.12	7.63/4.21/4.50
Spatial resolution (QClear)		
1 cm radial/tangential/axial FWHM (mm)	1.86/2.38/3.16	1.62/2.30/3.09
10 cm radial/tangential/axial FWHM (mm)	1.86/1.93/2.99	1.94/2.01/3.36
20 cm radial/tangential/axial FWHM (mm)	1.69/2.60/3.16	1.87/2.09/3.32
Spatial resolution (FFBP)		
1 cm radial/tangential/axial FWHM (mm)	4.50/4.27/4.96	4.25/4.21/5.02
10 cm radial/tangential/axial FWHM (mm)	5.57/4.60/5.80	5.58/4.56/6.40
20 cm radial/tangential/axial FWHM (mm)	7.45/4.99/6.14	7.50/4.98/6.74
Sensitivity		
At center (cps/kBq)	22.01	32.64
10 cm (cps/kBq)	22.59	32.88
Count rate statistics		
Peak NECR (kcps)	312.9	434.3
Peak NECR activity (kBq/mL)	22.5	23.6
Scatter fraction at peak NECR (%)	41	40.21
Maximum error at peak NECR (%)	4.61	3.95
Maximum mean error at peak NECR (%)	2.31	2.77
Image quality (VPFX)		
10 mm (CR (std)/BV (std); %)	48.0 (5.4)/8.5 (0.5)	54.5 (5.5)/6.8 (0.5)
13 mm (CR (std)/BV (std); %)	57.8 (7.7)/6.8 (0.3)	63.2 (3.2)/5.0 (0.6)
17 mm (CR (std)/BV (std); %)	70.4 (2.6)/5.2 (0.4)	68.0 (1.5)/4.0 (0.6)
22 mm (CR (std)/BV (std); %)	78.2 (1.3)/3.8 (0.5)	76.9 (4.0)/3.2 (0.3)
28 mm (CR (std)/BV (std); %)	83.3 (1.7)/2.8 (0.3)	82.4 (1.25)/2.5 (0.3)
37 mm (CR (std)/BV (std); %)	86.9 (1.3)/2.3 (0.1)	85.8 (1.6)/1.9 (0.2)
Average lung error (std) (%)	4 (0.2)	3.16 (0.1)
Image quality (QClear)		
10 mm (CR (std)/BV (std); %)	64.5 (3.5)/6.5 (0.4)	66.4 (3.0)/4.6 (0.4)
13 mm (CR (std)/BV (std); %)	70.7 (4.6)/5.2 (0.3)	72.5 (2.4)/3.5 (0.5)
17 mm (CR (std)/BV (std); %)	78.3 (1.7)/3.7 (0.4)	77.7 (0.5)/2.5 (0.5)
22 mm (CR (std)/BV (std); %)	84.2 (0.8)/2.8 (0.4)	84.8 (1.4)/1.9 (0.2)
28 mm (CR (std)/BV (std); %)	88.6 (0.8)/2.2 (0.2)	87.4 (0.7)/1.5 (0.2)
37 mm (CR (std)/BV (std); %)	90.2 (0.7)/1.8 (0.1)	89.6 (0.9)/1.3 (0.1)
Average lung error (std) (%)	3.2 (0.1)	2.6 (0.1)
Timing and energy resolution		
Energy resolution (%)	9.68	9.63
Timing resolution at 0 kBq/mL (ps)	391.6	389.6
Timing resolution at 5.3 kBq/mL (ps)	408.5	407.6
Scatter fraction		
Fraction (%)	41	40.21
Co-registration accuracy		
Error at (0,1) (5 cm/100 cm) (mm)	2.93/3.25	1.35/2.21
Error at (0,20) (5 cm/100 cm) (mm)	2.65/3.16	1.82/2.30
Error at (20,0) (5 cm/100 cm) (mm)	2.76/3.21	1.82/2.59

**Fig. 4:**
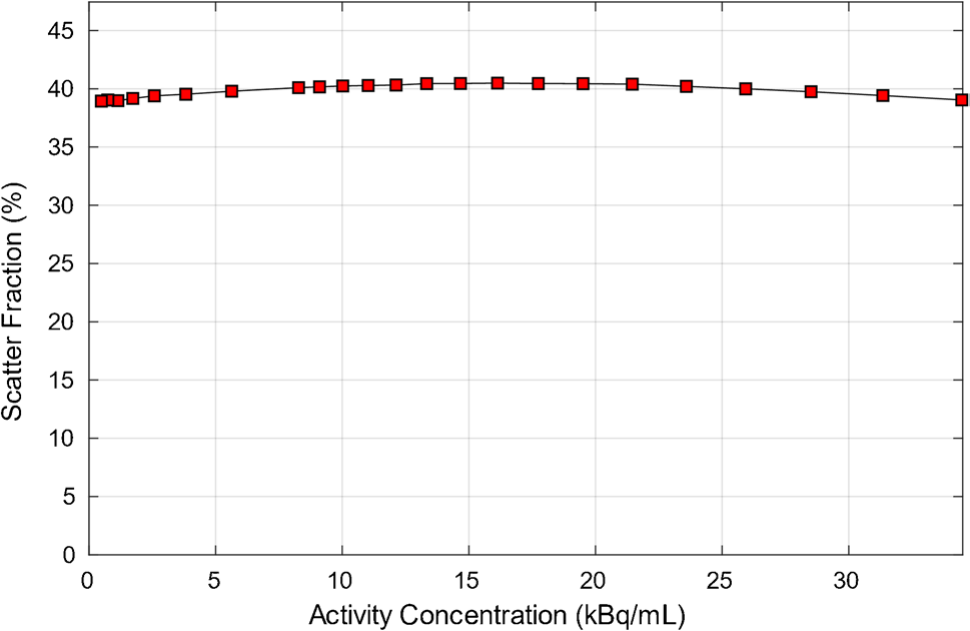
Scatter fraction for the 6-ring Gen2 DMI system at various activity concentration levels. Scatter fraction at peak NECR was found to be 40.21%. Due to similarity between system configurations, only the 6-ring system results are presented

**Fig. 5:**
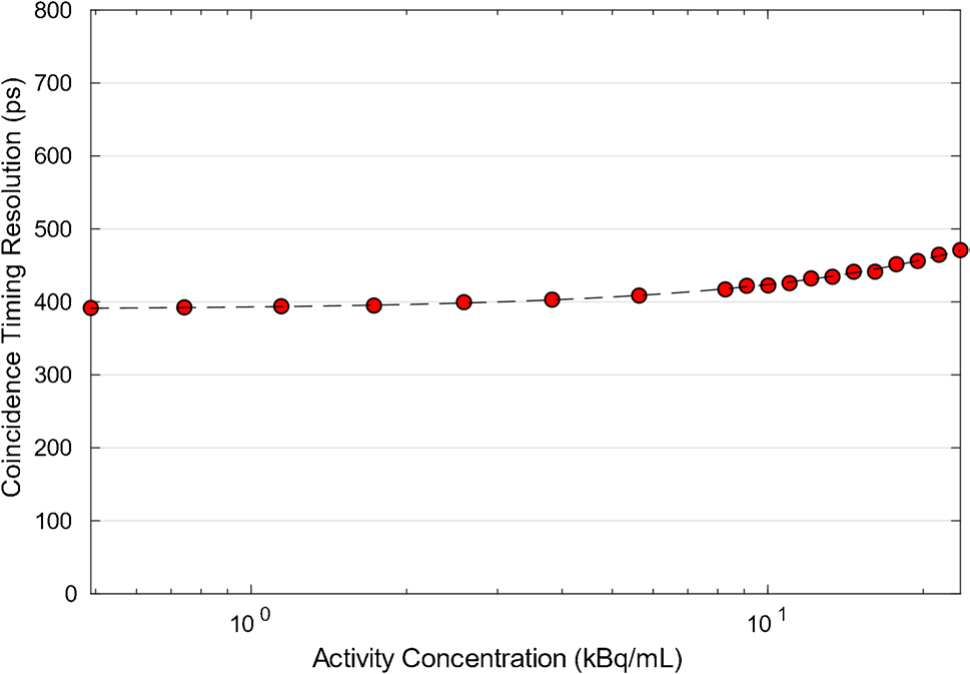
Coincidence timing resolution for the 6-ring Gen2 DMI system at various activity concentration levels. Timing resolution was measured at 389.6 ps and 407.6 ps at 0 and 5.3 kBq/mL, respectively. Due to similarity between system configurations, only the 6-ring system results are presented

Scatter fraction (40.21% and 41%) and energy resolution (9.63% and 9.68%) were very similar between the 2 configurations (Fig. 4). Timing resolution was found to be 389.6 ps and 407.6 ps at 0 and 5.3 kBq/mL respectively for the 6-ring Gen2, while for the 5-ring Gen2, it was measured at 391.6 ps and 408.5 ps at 0 and 5.3 kBq/mL, respectively (Fig. 5). PET/CT co-registration accuracy was 1.35 mm, 1.82 mm, and 1.82 mm at 5 cm and 2.21 mm, 2.30 mm and 2.59 mm at 100 cm, and lower to the 5-ring system (2.93 mm, 2.65 mm and 2.76 mm at 5 cm and 3.25 mm, 3.16 mm, and 3.21 mm at 100 cm). Measurements are summarized in Table 1.

### Clinical imaging

**Fig. 6:**
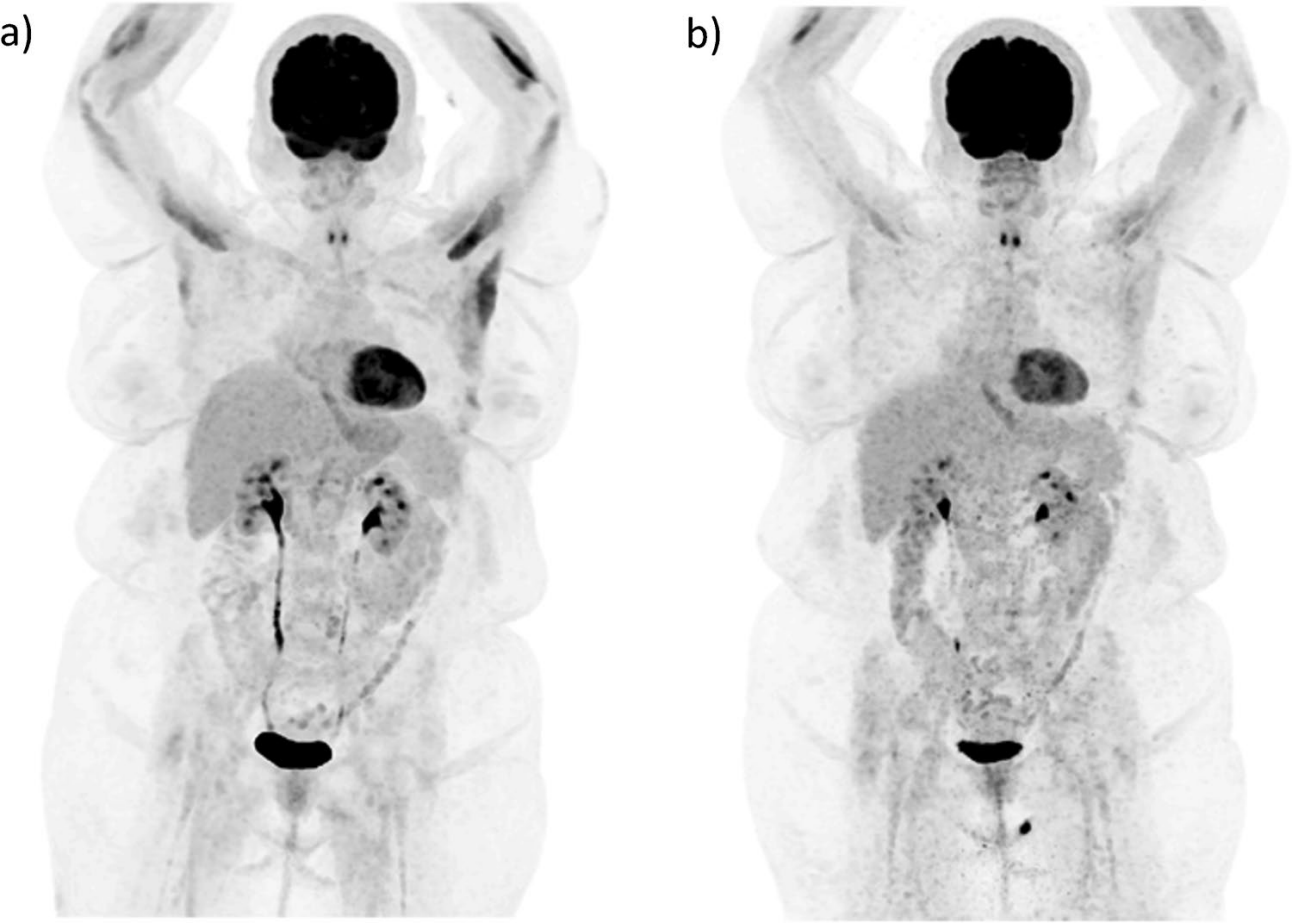
Example ^18^F-FDG patient (~ 2.6 MBq/kg and 10-min total scan time in both systems) acquired on the 6-ring Gen2 DMI (**a**) and on the 5-ring configuration (**b**) of the same system, reconstructed with QClear (beta 450) and data-driven respiratory motion correction (MotionFree, GE Healthcare)

**Fig. 7:**
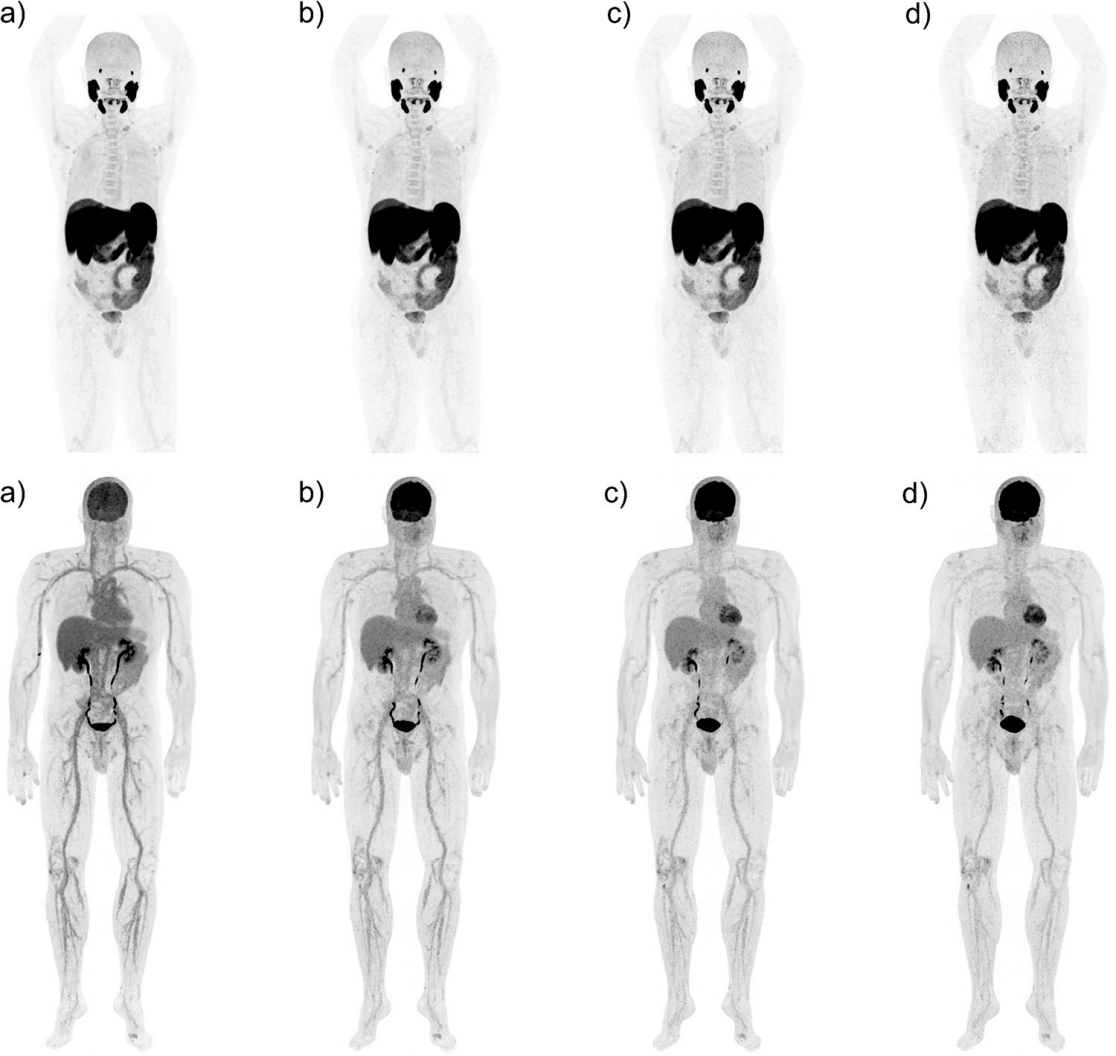
Example 6-ring DMI Gen2 ^18^F-PSMA patient (3 MBq/kg), reconstructed with QClear (beta 450) at 150 s/bed (**a**), 120 s/bed (**b**), 90 s/bed (**c**), and 60 s/bed (**d**) (top row), and ^18^F-FDG patient acquired at 11 min (**a**), 25 min (**b**), 39 min (**c**), and 54 min (**d**) post-injection with a dedicated WB dynamic protocol (bottom row)

Figure 6 shows an example patient scanned both on the 5-ring and 6-ring Gen2 DMI. Despite the fact that data were acquired at 2 separate sessions, all injection and acquisition parameters were comparable allowing for qualitative assessment. Looking at the maximum intensity projection images, the one acquired with the 6-ring system appears less noisy throughout the torso, especially when comparing across the liver where activity concentration is uniformly distributed. In Fig. 7A, a patient dataset was replayed at ever decreasing scan times demonstrating the ability to utilize the improved sensitivity of the 6th ring to drive overall scan time reduction in static acquisitions. Such scan time reduction per single static pass allows the acquisition of multiple WB dynamic frames as demonstrated in Fig. 7B.

## Discussion

In this work, we measured the performance of a new generation Discovery MI PET/CT system, which features, among other things, improved detector electronics and the ability of the detector modules to house a 6th detector ring, extending the maximum possible axial FOV to 30 cm. The system installed at our institution operated clinically as a 5-ring configuration prior to being upgraded to a 6-ring configuration. This fact allowed for a direct comparison between the 2 detector configurations of the same system, both from a technical and clinical perspective. The additional 6th ring not only extended the axial FOV to 30 cm but also resulted in an almost 47% increase in intrinsic sensitivity over the 5-ring configuration. This absolute sensitivity increase is in line with a theoretical quadratic increase with the axial FOV at small solid angles such that 6-ring sensitivity = 5-ring sensitivity × (6/5)^2^. Using then the average measured 5-ring sensitivity (22.3 cps/kBq), one could anticipate a theoretical sensitivity of 32.11 cps/kBq which is within 2% of the measured one at 32.76 cps/kBq. At substantially higher axial FOV, the increase in absolute sensitivity is expected to approach a linear relation to the axial FOV. This is due to the point source sensitivity at the center of the axial FOV reaching a plateau as the solid angle reaches the limit for a given ring diameter. Comparing across previously published data on lower ring configurations of the same system, the 6-ring sensitivity represents an almost 340% increase over the 3-ring configuration at 7.5 cps/kBq, with the doubling of the axial FOV resulting again in an almost quadratic change in sensitivity, and almost 140% over the 4-ring one at 13.7 cps/kBq [10]. This increased system sensitivity can be used to improve image quality, reduce scan time, and reduce injected activity or any combination of the aforementioned, in static imaging applications. However, it can also be used to enable multi-bed dynamic acquisitions, towards time activity curve assessment or/and kinetic parameter estimation, which are inherently more count limited compared to traditional single-bed dynamic acquisitions, owning to temporal gaps between frames. In the context of dynamic imaging, alternatively one can opt for increased temporal sampling depending on the radiotracer used and given a fixed total scan time, or maintain all other variables and reduce the overall scan time of the dynamic scan depending on the foreseen application. This is particularly of interest if such multi-bed dynamic protocols are to be used in clinical practice as opposed to clinical research.

The extended scanned axial FOV by the additional 6th ring enabled data acquisition with less bed positions, improving patient comfort, particularly in head-to-toe examinations. Furthermore, it allows almost the entire thoracic region to be included within a single-bed axial FOV, which could result in reducing the average number of beds being triggered for data-driven respiratory motion correction. Looking at the sensitivity of the 6-ring DMI Gen2 with systems of comparable FOV, the thicker crystal of the GE Discovery MI system (25 mm) results in much higher sensitivity per axial unit length at 1092 cps/MBq/cm [11–13].

Apart from the axial FOV and corresponding sensitivity increase, the improved dmod electronics in combination with the added ring resulted in an almost 60% improvement in peak NECR over the 5-ring Gen1 system, 140% over the 4-ring system (181.3 at 20.6 kBq/mL), and 335% over the 3-ring system (100 kcps at 20.6 kBq/mL) [3, 10].

The optimal activity concentration at peak NECR is also increased slightly potentially due to the fact that larger part of the phantom is within FOV, reducing the out of FOV randoms and increasing trues. Furthermore, the activity at which peak NECR occurs was also ~ 14% higher. The impact of the improved photon-counting electronics becomes more apparent when comparing peak NECR of our Gen2 5-ring system with reported Gen1 5-ring peak NECR, resulting in ~ 15% improvement with the peak NECR appearing at ~ 8% higher activity concentration [3]. Such improvements can be of importance more so in dynamic studies and here particularly in early frames following bolus injections due to high localized count rates, where accurate activity concentration both in the tissue as well as in the blood pool (when image-derived input functions are extracted) are needed. Going from 25- to 30-cm axial FOV results in clear improvement in peak NECR. However, at larger axial FOV configurations, any further increase could be penalized by the increased random contribution due to the necessity for a wider coincidence time window to accommodate ever more oblique LORs, as well as by the ever-increasing attenuation of the true coincidences at higher acceptance angles and increased scatter and random fractions. The axial length though, at which diminishing returns are obtained unless limiting the maximum ring difference, has been reported previously to be at axial lengths > 50–60 cm, though with TOF, it could be at higher axial lengths [10]. Hence, until such an axial FOV is reached, continuous peak NECR improvements are expected, with those progressively less obvious at ever-increasing axial FOV.

Contrast recovery values across all spheres were similar across the 2 Gen2 configurations tested in this work as well as with previously reported values from Gen1 systems from all configurations [6, 7, 9, 14]. This is true also for scatter fraction, as well as energy, spatial, and time resolution with broadly similar values across the 4 different ring configurations. However, as expected, background variability is substantially improved going from the 3-ring to the 6-ring configuration as a result of the ever-increasing point source and absolute system sensitivity.

In conclusion, we evaluated the performance of a new generation 30-cm 6-ring Discovery MI PET/CT system according to the NEMA NU-2 2018 standard. This system was shown to provide 47% higher sensitivity and 60% higher peak NECR compared to the 5-ring Gen1 system, while the rest of the image quality parameters were comparable among configurations. The higher sensitivity performance may clinically translate into reduced administered activity/reduced acquisition time, may improve image quality, or may enable dynamic imaging beyond single-bed positions towards multi-bed acquisitions.
